# Point Cloud Generation from Aerial Image Data Acquired by a Quadrocopter Type Micro Unmanned Aerial Vehicle and a Digital Still Camera

**DOI:** 10.3390/s120100453

**Published:** 2012-01-04

**Authors:** Tomi Rosnell, Eija Honkavaara

**Affiliations:** Finnish Geodetic Institute, Geodeetinrinne 2, 02430 Masala, Finland; E-Mail: eija.honkavaara@fgi.fi

**Keywords:** unmanned aerial vehicle, photogrammetry, point cloud, small sensor digital camera, calibration

## Abstract

The objective of this investigation was to develop and investigate methods for point cloud generation by image matching using aerial image data collected by quadrocopter type micro unmanned aerial vehicle (UAV) imaging systems. Automatic generation of high-quality, dense point clouds from digital images by image matching is a recent, cutting-edge step forward in digital photogrammetric technology. The major components of the system for point cloud generation are a UAV imaging system, an image data collection process using high image overlaps, and post-processing with image orientation and point cloud generation. Two post-processing approaches were developed: one of the methods is based on Bae Systems’ SOCET SET classical commercial photogrammetric software and another is built using Microsoft^®^’s Photosynth™ service available in the Internet. Empirical testing was carried out in two test areas. Photosynth processing showed that it is possible to orient the images and generate point clouds fully automatically without any a priori orientation information or interactive work. The photogrammetric processing line provided dense and accurate point clouds that followed the theoretical principles of photogrammetry, but also some artifacts were detected. The point clouds from the Photosynth processing were sparser and noisier, which is to a large extent due to the fact that the method is not optimized for dense point cloud generation. Careful photogrammetric processing with self-calibration is required to achieve the highest accuracy. Our results demonstrate the high performance potential of the approach and that with rigorous processing it is possible to reach results that are consistent with theory. We also point out several further research topics. Based on theoretical and empirical results, we give recommendations for properties of imaging sensor, data collection and processing of UAV image data to ensure accurate point cloud generation.

## 1. Introduction

In recent years unmanned aerial vehicles (UAVs) have entered the field of aerial imaging. Low operation and hardware costs with low altitude UAVs compared to the aerial imaging with high quality mapping sensors from manned airborne platforms have made UAVs an attracting choice for aerial photogrammetry in many application areas [1,2]. The systems are very competitive in local area applications and especially if repetitive data collection or rapid response is needed. Emerging application areas include various monitoring applications, such as monitoring of agricultural crop [2–5], geometric changes of environment such as rockslide or volumetric changes of gravel pits [2], archaeological sites [2,6], disasters [7–10] and traffic [11].

An important recent cutting-edge advancement in the photogrammetric processing is the comeback of image matching for digital elevation model generation [12]. There already exist several new approaches for high-quality, dense point cloud generation from passive image data by image matching [13–16]. The image based point clouds can be utilized in a manner similar to that laser scanner based point clouds are utilized, or they both can be utilized together. Image based point clouds are an attracting alternative for laser scanning, because they enable high precision 3D mapping applications for low-cost and low-weight systems. Laser scanners, which require high precision Global Navigation Satellite System and inertial measurement unit (GNSS/IMU), cannot be operated currently from very low-weight UAV platforms [17,18].

Since 2009, the Finnish Geodetic Institute (FGI) has been operating light UAV imaging systems consisting of quadrocopter type micro UAVs and small-format cameras. Our objective is to develop methods for local area remote sensing and monitoring with these systems. A recent study of reflectance calibration of UAV image data showed that good performance can be obtained with careful processing [19]. A specific objective in this study was to develop and investigate methods for point cloud generation. Characteristic features of the data collected by the light quadrocopter UAVs, which have unstable sensors and imaging platform, include large numbers of images that typically have large rotational differences and poor quality direct orientation observations; also non-conventional imaging geometries with vertical and oblique images are very feasible. Processing of this complicated data is much more challenging than processing of image data collected using robust airborne mapping cameras in regular block set-ups. While investigating data collection, orientation and point cloud matching processes, we developed two processing methods for point cloud generation with these systems.

The content of this article is as follows. The system for image matching based point cloud generation is described in Section 2. Set-up of empirical testing is described in Section 3 and results are given in Section 4. The summary and recommendations are given in Section 5.

## 2. Description of the System

The major components of the point cloud generation system are a UAV imaging system, a data collection process and a post-processing phase consisting of orientation of images and point cloud generation. In the following, the system that was developed at FGI is described.

### 2.1. Quadrocopter Based UAV Imaging Systems

**Figure 1.**
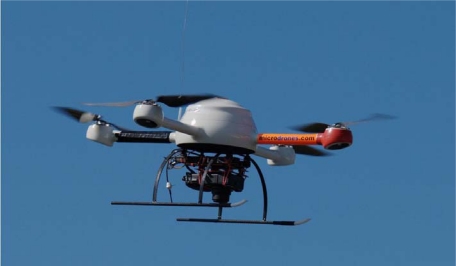
Microdrone md4-200 with a Ricoh GR3 compact camera.

Two quadrocopter UAV based imaging systems have been developed. The first is based on a Microdrone md4-200 (Figure 1), which is an electronic battery-powered quadrocopter UAV manufactured by Microdrones, Germany [20] and is able to carry a 300 g payload. The second system is based on a Microdrone md4-1000, which is a larger version of the md4-200 that can carry a 1.2 kg payload. Both types of UAV can take off from and land vertically on a small open area and have an onboard flight controller with a compass and inertial, gyroscopic, barometric and GPS sensors. The flight times are with the current batteries 10–20 min for the md4-200 and 20–30 min for the md4-1000. The systems are sensitive to wind, and lower than a 4 m/s wind is needed to obtain a controlled image block. Wind speeds over 4 m/s tilts these UAVs drastically and leads to large pitch and roll angles.

The md4-200 used in the study was equipped with a Ricoh GR Digital III (Ricoh GR3) digital compact camera [21]. The camera has a lens with a 6 mm fixed focal length and f-stop values of 1.9–9. The camera holds a 7.6 mm × 5.7 mm (3,648 × 2,736 pixels) CCD sensor with a pixel size of 2 μm. The weight of the camera is 180 g, excluding the battery and the memory card. With md4-1000, Panasonic Lumix GF1 (Lumix GF1) was used. It has a Live Mos sensor of the size of 17.3 mm × 13.0 mm (4,000 × 3,000 pixels) with a pixel size of 4.5 μm [22]. GF1 was equipped with a lens with a fixed 20 mm focal length that had f-stop values of 1.7–16. The total weight of the camera is 448 g. With both systems, the raw images are stored and developed to RGB images using the free-ware DCRAW-software [23]. A radiometric processing line for md4-200 is developed in [19].

### 2.2. Development of Image Capturing Modes

The two elementary image capturing modes the systems have are the preprogrammed flight mode and the manual flight mode. We have evaluated their feasibility in our applications.

We tested two approaches based on the preprogrammed flight mode. First of all, we tried a preprogrammed flight with preprogrammed image capturing spots. In this approach, Microdrone stops to hover in a fixed position to shoot an image. We have found this approach impractical for mapping purposes, because stopping to hover in a fixed position and accelerations before and after the stops take a lot of time. Thus, if one wants to take images with a high overlapping percentage, the flight distance becomes very short. Moreover, in our experience when Microdrone tries to hover in a fixed position, it becomes very unstable. Another approach is to carry out the preprogrammed flight route without stopping for image capturing but setting the camera to continuous or interval shoot mode. With this approach, it is possible to travel a great deal more distance than with stops and the system is more stable.

Two image collection approaches based on manual flight mode were evaluated. The first approach was basic flying where we tried to visually estimate the location of the UAV. With this approach, the camera was always aimed straight down. Another approach we used was flying with the aid of live camera feed and virtual glasses showing the image from the camera on the UAV. In this case, we were able to aim every shot independently and control the camera’s pitch angle. Both manual approaches are highly dependent on the skill level and experience of the pilot controlling the UAV. The main advantage of the manual flight mode over the preprogrammed flight mode comes with flight speed. Microdrone allows a greater flight speed with the manual flight mode than with the preprogrammed flight mode. With different manual approaches, we have used a continuous or interval shoot for the camera.

### 2.3. Geometric Processing

Geometric processing involves determination of the image orientations and the point cloud generation. In the rigorous photogrammetric processing, the imaging model based on the collinearity condition is used [24–27]. The model parameters include the exterior orientation parameters (perspective center coordinates and rotations of the image; X0, Y0, Z0, ω, φ, κ) and the camera parameters, including the interior orientations (principal point and principal distance; x0, y0, c) and image distortions. A popular distortion model for digital still cameras is the physical model with radial distortion (parameters: k1, k2, k3), asymmetric distortion (parameters: p1, p2) and in plane distortion (parameters: b1, b2) (see details of models in [24]). Furthermore, in integrated systems also lever arms and boresight angles between individual systems have to be determined [28].

The determination of orientations of images collected using airborne mapping cameras has reached a high performance level [29–31], but the orientation of UAV images is more challenging due to the diversity of the data. A popular approach is to use an integrated sensor orientation, which determines the parameters in a combined adjustment with direct sensor orientation observations, tie points and ground control points (GCPs). Integrated approach is necessary in order to obtain accurate results, because the high-accuracy GNSS/IMU systems cannot be operated (currently) with low payload UAVs due to weight restrictions, and also the calibration of the system (alignment of the camera and IMU, camera parameters) can be unstable [8–10,32,33]. If the accuracies of the exterior orientations measured by the GNSS/IMU are low, they serve mostly as approximate orientation values. Despite the challenges, a good performance level has been reported for the approaches based on integrated sensor orientation [2,8–10,34,35].

Important new innovations for orientation determination include the approaches which do not require any a priori orientation information. These methods are capable of automatically handling unordered image collections captured using different non-calibrated cameras, in different lighting conditions and in different perspectives [36,37]. Examples of operational applications or services based on these techniques are Microsoft’s Photosynth [38] and Geosynth [39]. These applications apply matching techniques that are invariant to dissimilarities between images, such as the SIFT operator and its variants [40,41] and the SURF operator [42]. Excellent performance of these approaches has already been proven in orientation of UAV images [41,43,44].

Different matching methods can be used for point cloud generation [12–16,40–42,45–50]. In photogrammetric applications, often signal based matching methods (least squares matching, cross correlation) are used; recently, a new semiglobal matching method has shown very promising results [16]. A high performance level, with high success rations, good penetration in 3D objects, and high accuracy, is reached by using large image overlaps and matching multiple images in each point [12–16,48–50].

The objective was to construct the system using commercially available or freeware components as far as possible. The phases of our process are terrestrial calibration of cameras by the Photometrix iWitness AutoCal software [51] and post-processing after image collection with orientation determination and point cloud generation. We developed two post-processing approaches: one is based on BAE Systems’ SOCET SET, a conventional commercial photogrammetric software, and the other one on the Microsoft^®^ Photosynth™ service available in the Internet; both are augmented with in-house components.

#### 2.3.1. Photosynth Processing

The Internet freeware processing is based on the Microsoft^®^’s Photosynth™ service [38]. Photosynth automatically stitches a set of photos taken of the same scene or object into one interactive 3D-viewing experience that can be shared with anyone on the web. Photosynth combines two operating devices in one product: a viewer for downloading and navigating complex visual spaces and a synther for creating them. The synther is based on feature-based matching techniques that are less sensitive to geometric and radiometric differences between images than traditional matching techniques [40]. The synther process provides a 3D-shape of the subject (point cloud) and the interior and exterior orientations of each photo used.

When using Photosynth, we first of all determine the sensor calibration using iWitness, and undistort all images. Then we load the undistorted JPEG-compressed images to the Microsoft’s server and launch the Photosynth process. The data provided by Photosynth are exported to a local PC with SynthExporter [52]. The exported data contains the exterior orientations as well as some camera parameters (principal distance, two radial distortions (k1, k2)) for every image and the point clouds with X, Y and Z coordinates and red, green and blue color values. Photosynth performs the processing in its internal coordinate system. We use accurate and well-identifiable control points or objects to transform the Photosynth point cloud to a desired object coordinate system, in our case the ETRS-TM35FIN. A 7-parameter similarity transformation is used to carry out the coordinate system conversion. The initial values for rotations are calculated with the singular value decomposition, and the final solution is obtained with the least squares adjustment method.

#### 2.3.2. SOCET SET Processing

The photogrammetric processing is based on the BAE Systems’ SOCET SET commercial photogrammetric software [53]. The software follows rigorous photogrammetric processing methods and provides the means for performance analysis by various statistical tools [25,26].

The orientations of TIFF-images are determined using the Multi Sensor Triangulation module (MST). We aim to a large number of automatically measured tie points, and the control points are measured interactively. After the point measurements, self-calibrating bundle block adjustment is carried out. Physically based distortion models (see above) are then used to model image distortions. Direct observations of exterior orientations (GNSS or GNSS/IMU) can be utilized in MST either by carrying out direct georeferencing or integrated sensor orientation [28,53].

Next Generation Automated Terrain Extraction software (NGATE) is used to generate three dimensional point clouds [48,49]. NGATE has been proven to provide dense and accurate point clouds from large-format mapping cameras [54]. The matching strategy employs the edge and correlation matching methods and applies image pyramids and back matching. Various pre-defined matching strategies are available in the NGATE software.

### 2.4. Expected Performance

Quality of a point cloud can be characterized by two major quality indicators: the completeness of the point cloud and accuracy of individual points. Further aspects are the quality indicators of elevation products, which are not considered in this investigation [55].

In the point determination, the accuracy of an object coordinate (σ_obj(X,Y,Z)_) can be presented as a function of two major error components [25]:

(1) $$\sigma_{\text{obj}(X,Y,Z)}=\sqrt{\sigma_{\text{isec}(X,Y,Z)}{}^{2}+\sigma_{\text{def}(X,Y,Z)}{}^{2}}$$

Intersection error σ_isec(X,Y,Z)_ is dependent on geometric factors, mainly on the orientation accuracy and point intersection geometry. Object definition error σ_def(X,Y,Z)_ is the deviation caused by positioning error due to matching imprecision.

The completeness of the point cloud is dependent on the block overlaps (especially occlusions) and on the performance of the matching method.

The performance of the matching method is thus the fundamental factor influencing both the accuracy and the point density. It is mostly dependent on image quality, object and matching method related factors as follows:

1. Central factors influencing the image quality include the sensor quality (signal-to-noise ratio), the imaging system (especially image motion) and the atmospheric, illumination and wind conditions during the data collection.
2. As is well known from conventional stereoscopic measurements and image matching, different performance is obtained with different objects [25,49]. Typically, difficulties are experienced with thin objects (such as electric poles), vague objects (for example, trees), homogeneous objects, repetitive features and water surfaces.
3. The matching method and its radiometric and geometric assumptions have a great influence on the quality of matching [45,46]. Signal-based matching methods are known to be more accurate than feature-based matching methods, but less tolerable for differences in images. We expect that a better accuracy is obtained with the SOCET SET process than with the Photosynth process, because SOCET SET utilizes a more accurate matching method.

Factors (1–3) are not independent; for example, we expect that the performance obtained for a homogeneous object in good imaging conditions is different from that obtained in poor imaging conditions. There are also differences in the results when the image collection is done with a high quality sensor as compared to it having been done with a low quality sensor. Theoretical relationships are derived in [54].

## 3. Empirical Study

### 3.1. Flight Campaigns

Missions were carried out in two test areas: the Sjökulla test site and the FGI main building in Masala. The major analysis was carried out using the Sjökulla data, while the objective of the Masala mission was to evaluate general performance of point cloud generation in a built area with large height differences, using different imaging system and in different imaging conditions.

#### 3.1.1. Sjökulla Mission by md4-1000

FGI is maintaining a photogrammetric test site at Sjökulla (60°14′31″, 24°23′1″) [56]. The UAV mission was carried out in Sjökulla on 24th June 2010 at 11:00–12:00 local time in a small area of about 300 m × 300 m in size. The imaging conditions were excellent with a clear sky, a high solar elevation of 51–53° and practically no wind. UAV Microdrone md4-1000 with the Lumix GF1 camera was used. The flight routes were planned with the Microdrone’s md-cockpit software. Camera settings were on manual during the flights and the lens was focused to infinity. ISO sensitivity 200 was used to minimize image noise. The aperture was set to 2.2, and the shutter speed was 1/3,200 s. Fast shutter speed was used to minimize image blurring caused by platform vibrations and on-flight movement.

**Figure 2.**
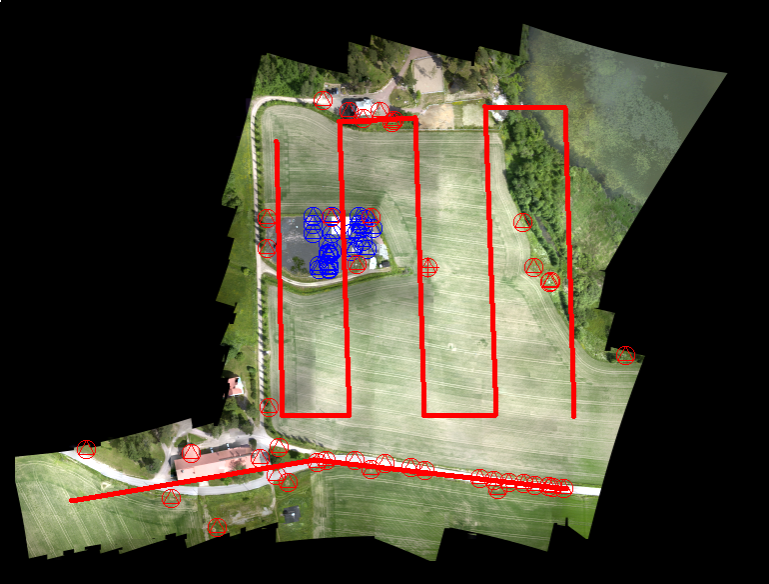
Flight routes of the reduced image block that was used in the SOCET SET processing. GCPs are in red and checkpoints are in blue.

The entire dataset contained a total of 251 images in 24 image strips, out of which some were overlapping 100% and some were transfer flights from one point to another. Images were collected from a flying height of 110–130 m above the ground level, providing a ground sampling distance (GSD) of 2.4–2.8 cm on average. The forward overlaps in image strips were 80–90% and the side overlaps were 50–100%. In the Photosynth processing all the images were used. In the SOCET SET processing a reduced data set with 122 images was used, with five lines in North to South direction and one line in West to East direction following an asphalt road in the southern part of the area (Figure 2). In this data, the overlaps were 80–90% in the flying direction and 50% between the image strips, on average.

The flight area at the Sjökulla test site contains an image quality test site, fields, trees, buildings and roads with gravel and asphalt coatings. In the UAV mission area, altogether 11 targeted GCPs were available (accuracy of 1 cm in planimetric coordinates and 2 cm in height). Additional control points were derived using image data collected in October 2009 by the UltraCamXp large-format photogrammetric mapping camera [57] (two image strips with 60% forward and 30% side overlaps, flying height 740 m, GSD 4.2 cm). Estimated accuracy of the photogrammetric control points was 2 cm in planimetric coordinates and 6 cm in height. Altogether 31 GCPs were used, mostly located in the ends of the image strips and in the block perimeters. All of the 24 checkpoints were located in the central area of the image block. Reference digital surface model (DSM) with 10 cm point interval was derived using the NGATE with an estimated height accuracy of 6 cm for well-defined objects. There is a temporal difference between the reference and the UAV campaign data. It is expected that the UltraCamXp reference is accurate for many targets, such as buildings, roads and test site (in the test site, frost might have caused some changes, however). Field surface (ploughing in spring, crop) and other areas covered by vegetation (trees, bushes, grass) are less stable targets.

#### 3.1.2. Masala Mission

**Figure 3.**
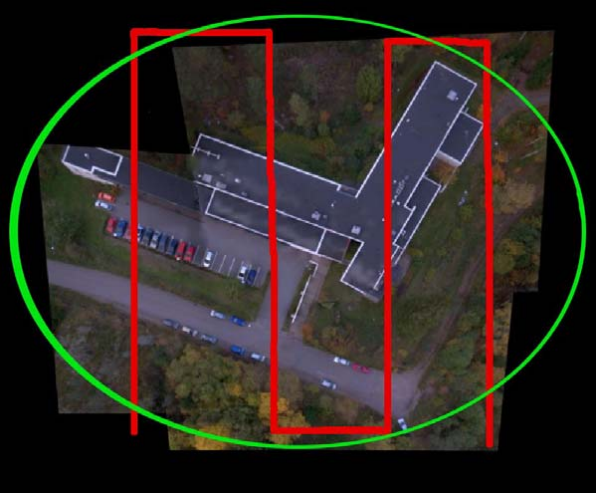
Flight routes in the Masala mission. The regular block with vertical images is in red and the rotating image flight with oblique images aimed at the building is in green.

The second mission was carried out in the surroundings of the main building of FGI (60°9′39″, 24°32′41″) (Figure 3). The building has a flat, black felt-covered roof. The area extends to a sparse forest in the north, the ground surfaces south from the building are mainly covered with asphalt, and in other sides there are separated trees and grass cover. A total of 14 building corners and road paintings were used as GCPs. They were measured by GNSS using virtual reference stations (VRS) with an approximate accuracy of 3 cm.

The mission was carried out on 30th September 2010 at 11:00–12:00 local time. The imaging conditions were weak, as the sky was fully covered by clouds; the solar elevation was about 27°. The imaging system with Microdrone md4-200 and Ricoh GR3 were used. Two different flight modes were employed. The first flight mode was a continuous shoot with pre-programmed flight lines in North-South directions. The second fight mode was a continuous shoot with a manual flight control. During the manual flight, the camera was pointed to the building while the flight went around the building. Live video feed from the camera and virtual glasses were used for visual navigation. The camera settings were on manual during the flights, and the lens was focused to infinity. ISO sensitivity 100 was used to minimize image noise. The aperture was set to 2.8 and the shutter speed was 1/1,600 s. The entire dataset contained a total of 280 images in 7 image strips and 1 rotating circle. Images were collected from a flying height of about 70 m from the ground level, providing a GSD of 2.5 cm on average. The forward overlaps in the image strips were 75–90% and side overlaps 55–70%.

### 3.2. Performance Assessment

The quality of point clouds was evaluated based on the quality components described in Section 2.4 (Equation (1)):

1. The potential of the photogrammetric block was assessed by evaluating the quality statistics provided by the bundle block adjustment software (standard deviations of calculated orientation parameters and point unknowns) and by calculating the accuracy of point determination using independent checkpoints; the approach was developed in [27]. The point determination accuracy estimates correspond to σ_isec(X,Y,Z)_.
2. Height standard deviation in planar windows of size 3 m × 3 m was used to characterize the internal precision of point cloud matching. This is the estimate of σ_def(Z)_ for planar objects.
3. Absolute height error was evaluated by using the UltraCamXp point cloud as a reference. The point clouds were sampled to 1 m × 1 m grid by calculating the height value for each grid cell as an average of heights of the points in the cell; the differences of two point clouds were calculated for those grid cells that had height observations. This evaluation gives an estimate of σ_obj(Z)_. Average of height differences was used to characterize the systematic height error, and standard deviation of height differences to indicate random error. The differences that were larger than three times the standard deviation were considered as outliers and eliminated.
4. Relative point density (*r*) was used as the indicator of success rate of matching:
  (2) $$r={\mathrm{point\_density}}_{\mathrm{pc}}/{\mathrm{point\_density}}_{\mathrm{theor}}={\mathrm{point density}}_{\mathrm{pc}}/\left(1/{\mathrm{point interval}}^{2}\right)$$
  where *point_density_pc_* is the point density per square meter in the point cloud and *point_density_theor_* is the theoretical point density based on a selected point interval.

We studied theoretically the potential of the developed measurement system by simulation, for which the FGIAT bundle block adjustment software was used as described in [27]. The simulation was used to enable evaluation of the feasibility of various control scenarios and to obtain a reference calculation that is not influenced by possible systematic deformations in the data. Simulation is a basic technique used in developing photogrammetric measurement systems [58–60], but according to our knowledge it has not been used extensively in evaluation of UAV imaging systems.

## 4. Results

### 4.1. Simulation Study

**Figure 4.**
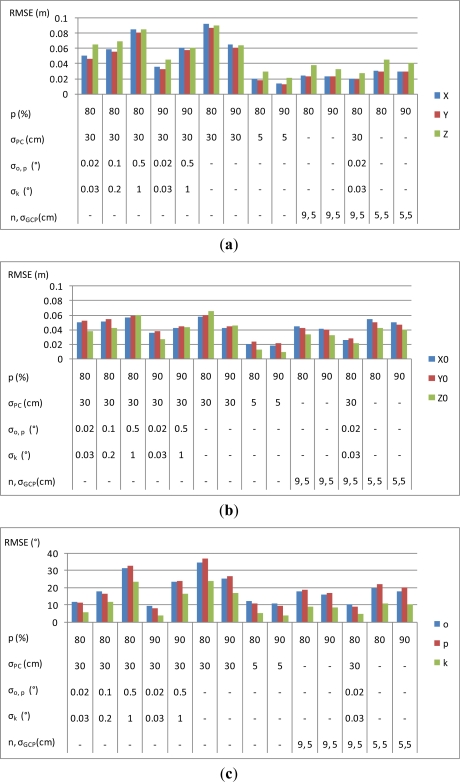
Statistics on simulations: standard deviations of (**a**) point determination, (**b**) positions of perspective centers and (**c**) image rotations. Settings that were used in simulations are shown below bars (p (%): forward overlap percentage; σ_PC_, σ_o,p_, σ_k_, σ_GCP_: standard deviations for perspective center, rotation and GCP observations; observations marked with ‘-’ were not included in simulation; o, p, k: ω, φ, κ; n: number of GCPs).

The block set-up was similar to the Sjökulla UAV mission (Section 3.1.1). The block had five parallel flight lines with a 60% overlap of the image strips, and the number of images per strip was 13 with an 80% overlap and 27 with a 90% image overlap in flight direction. Normally distributed random errors with zero mean were generated to the observations. Approximately 100 tie points were generated per image, with the image coordinate standard deviation of about pixel size/2 (2 μm). Calculations were carried out without GCPs, using nine uniformly distributed GCPs and using five GCPs (one in each block corner and block center), with a standard deviation σ_GCP_ = 5 cm (corresponding to the accuracy of state-of-the-art VRS GNSS positioning). Also the use of direct orientation observations was evaluated; the positions of the perspective centers had the standard deviations σ_PC_ = 5 cm and σ_PC_ = 30 cm (corresponding to the situations with high and medium quality GNSS positioning). Two scenarios were tested for standard deviations of image rotations: (1) accurate (σ_ω_ = σ_φ_ = 0.02° and σ_κ_ = 0.03°); and (2) low accuracy (σ_ω_ = σ_φ_ = 0.5° and σ_κ_ = 1°) [8]. The standard deviations of the solved point coordinates, image rotations and perspective center coordinates provided by the bundle block adjustment are shown in Figure 4.

The highest accuracy of about 1 cm in planimetric coordinates and 2 cm in the height coordinate was obtained by using accurate perspective center observations (σ_PC_ = 5 cm) and 90% forward overlaps. The use of nine GCPs with σ_GCP_ = 5 cm provided almost as high accuracy: 2 cm in planimetric coordinates and 3 cm in the height coordinate. With a lower quality of perspective centers (σ_PC_ = 30 cm) and no rotation observations, the accuracy was about 9 cm in all coordinates with 80% stereo overlap and 6 cm with 90% stereo overlap. Increasing the forward overlap from 80% to 90% clearly improved the accuracy of orientation parameters and point determination. This was because accuracy improves with the increasing number of observations, which is in accordance with the law of large numbers. Decreasing the number of GCPs decreased the accuracy. It is also remarkable that the use of the low accuracy rotation observations improved the accuracy statistics.

### 4.2. Calibration of UAV-Carried Cameras

Calibration was carried out by terrestrial test field calibration using iWitness and by self-calibration using SOCET SET. In iWitness we calibrated interior orientations (x0, y0, c) and radial lens distortions (k1, k2, k3). With SOCET SET we used greater number of parameters: interior orientation (x0, y0, c) and lens distortions (k1, k2, k3, p1, p2) (Section 2.3) (principal distance was used only for Masala data in SOCET SET). In SOCET SET, a single set of camera parameters for all images was used; alternatively, we could have estimated camera parameters for several subsets of images or separately for every image. The results of the calibrations are given in Table 1.

**Figure 5.**
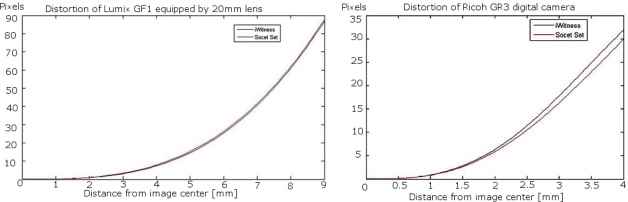
Radial distortions of Lumix (**left**) and Ricoh (**right**) cameras.

**Figure 6.**
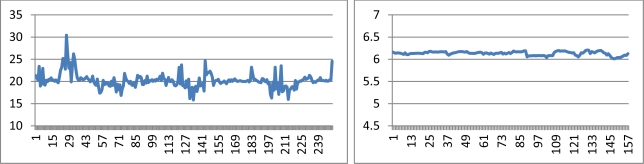
Focal lengths of Lumix GF1 (**left**) and Ricoh GR3 (**right**) for each image calculated by Photosynth (in mm).

The calibrations of the Lumix GF1 could be considered successful: the iWitness calibration provided a root mean square error (RMSE) of 0.17 pixels, while the standard deviation estimates provided by the SOCET SET processing indicated good calibration accuracy. iWitness and SOCET SET provided similar estimates of the effects of distortions, which were at their maximum 90 pixels (Figure 5). Principal point differed quite a lot in two calculations, which can be due to instability of the sensor or instability of the self-calibration solution or due to the fact that the camera has fallen down between two calibrations. For every image, Photosynth estimated the principal distance, which varied between 15.83 mm and 30.42 mm in different images. The average was 20.20 mm (Figure 6).

The iWitness calibration for the Ricoh GR3 was successful with a RMSE of 0.10 pixels. The SOCET SET provided relatively similar radial distortion parameters to those of iWitness. The radial distortion was about 35 pixels in the image corner (Figure 5). Photosynth calculated the principal distance, which varied between 6.01 mm and 6.21 mm, the average being 6.13 mm (Figure 6); the variation was clearly lower than in the Sjökulla block, which can be due to the better block geometry in Masala (a 3D object and vertical and oblique images).

### 4.3. Results of the Sjökulla Block

#### 4.3.1. General Results

**Figure 7.**
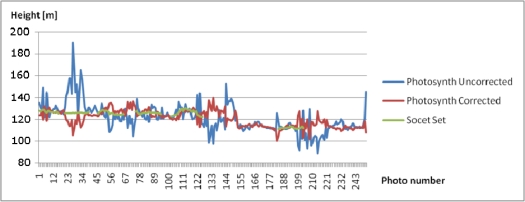
Heights of projection centers for the Sjökulla image block provided by Photosynth, corrected Photosynth and SOCET SET.

**Figure 8.**
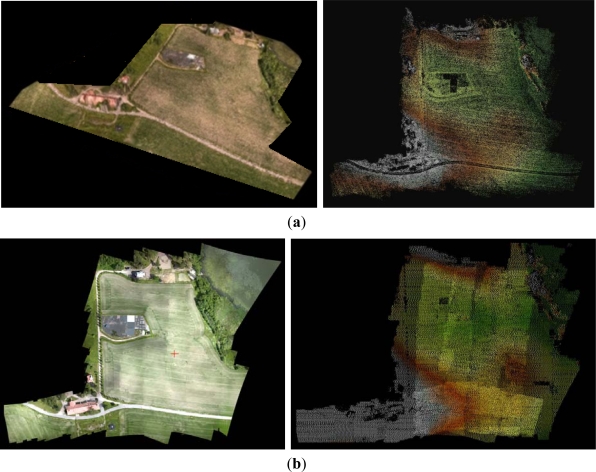
Point clouds from Sjökulla provided by (**a**) Photosynth (**left**: RGB, **right**: earth tones) and (**b**) NGATE (**left**: image mosaic, **right**: overlaid point clouds of image strips in earth tones). In point clouds the black areas are without points.

Photosynth was able to orient all images to a local coordinate system without any a priori orientation. The perspective center height values provided by Photosynth were correlated with the principal distance values, showing a variation between 100 m and 190 m, with the nominal flying height of 125 m, and a variation between 90 m and 140 m, with the nominal flying height of 110 m (Figures 6 and 7); SOCET SET provided a stable flying height. The flying heights were corrected using the relation between the calibrated focal length and the focal length by Photosynth, which reduced the height variation to 110–140 m and 100–130 m, respectively. The approach of treating each image as if it were from a different camera is well suited for photo collection applications or if zoom lenses and automatic focusing is used, but it is not ideal for applications using a single camera with a fixed focal length. Photosynth produced a RGB-colored point cloud in the local coordinate system (Figure 8(a)). Eleven targeted GCPs were identified from the point cloud and transformation to the ETRS-TM35FIN coordinate system was carried out. The RMSE after a 7-parameter similarity transform was 35.7 cm and the standard deviation of unit weight was 40.2 cm.

The SOCET SET processing included the orientation determination by MST and point cloud generation by NGATE. In the SOCET SET MST processing, 81 uniformly distributed tie points were measured automatically per image. We determined the initial approximations of exterior orientations by estimating the positions of image centers in an orthophoto. The statistics of the self-calibrating bundle block adjustment indicated high accuracy: standard deviations were 5 cm, 5 cm and 2 cm for the perspective center positions in the X, Y and Z coordinates, respectively, and 0.021°, 0.025° and 0.005° for the rotations ω, φ and κ, respectively (the calibration parameters are given in Table 1). The point cloud was created with NGATE by using every second image (stereo overlaps 60–80%) and three models per point (Figure 8(b)). We tested different matching strategies implemented in the NGATE software (ngate_urban, ngate, and ngate_urban_canyon). In this study, we selected for further evaluations the ngate_urban_canyon strategy that is intended for urban areas with large height differences, because it appears to provide the best accuracies and the best success ratio with the very large scale images. The most important parameter in this method is the correlation window size, which was 13 × 13 pixels.

#### 4.3.2. Point Density

We calculated point densities on different surface types in image windows of the size of 3 m × 3 m. A total of 119 sample squares were evaluated: 30 field, 24 forest, 30 grass, 20 asphalt road and 15 gravel road windows. Three different point clouds were evaluated: UltraCamXp point cloud by NGATE with a 10 cm point interval, GF1 point cloud by NGATE with a 5 cm point interval and Photosynth point cloud of GF1 images. The results are given in Table 2.

NGATE produced high quality point clouds with UAV images on even surfaces; the relative point densities were 0.7–0.9 for field, grass and gravel road; on asphalt road slightly lower densities appeared. For uniform objects, the success rate of the UAV point cloud was slightly lower than that of the reference UltraCamXp point cloud, which was most likely due to lower signal-to-noise-ratio in UAV images. Forests that had high three-dimensionality compared to flight altitude were more problematic: the relative point densities were under 0.5, even with images of the UltraCamXp, which occasionally had large empty areas. Sometimes single trees didn’t appear at all in point clouds. In the case of UltraCamXp, the 60% forward overlap was not ideal for matching in forest areas. These results show that the results of the UAV-carried small-format camera were comparable to a large-format photogrammetric camera in relative point densities for automatically measured point clouds. It is also possible that the NGATE parameters could be further tuned for large-scale images to obtain better results for 3D objects, but our trials did not improve the results.

The point density in the Photosynth point cloud was 2–3 points per m^2^ for all objects. Homogeneous objects (e.g., asphalt surfaces) caused more problems for Photosynth than for NGATE, which can be partially due to the fact that the Photosynth processing used JPEG-compressed images, which might have caused an additional decrease in the signal-to-noise ratio. On the other hand, Photosynth was more successful than NGATE in areas with vegetation and 3D objects.

#### 4.3.3. Height Accuracy of Point Clouds

First, we evaluated the point determination accuracy (estimate of σ_isec(X,Y,Z)_) of SOCET SET processing (Table 3). The RMSEs at independent checkpoints were 2.9 cm in X, 4.3 cm in Y and 10.6 cm in Z, and the standard error of unit weight was 0.87 pixels. The standard deviations of the differences were only slightly lower, which indicates that the block was only slightly distorted. Results of simulation with an image coordinate precision of 4.5 μm (1 pixel) (2.9, 2.7 and 6.2 cm) were similar to empirical results. When taking into account the inaccuracy factors of the photogrammetric checkpoints (Section 3.1.1), the accuracy of the UAV point determination with this data set was on the level of GSD in planimetric coordinates and better than 3 times GSD in height. We also carried out block adjustment without self-calibration; in this case the block was seriously distorted, and the RMSEs at independent checkpoints were 24 cm, 17 cm and 518 cm in X, Y and Z, respectively; the standard error of unit weight was 5 pixels. Theoretical standard deviations provided by bundle block adjustment were for all cases about 1 cm in planimetric coordinates and 3 cm in height. These estimates differ significantly from the empirical values, which is partially due to inaccuracy of checkpoints and systematic errors in the block which do not appear in standard deviations.

Internal precision of matching in flat objects (estimate of σ_def(Z)_) was studied by calculating the standard deviations of heights in 3 m × 3 m areas (Table 4). The height variation was about 10 cm on point clouds derived by NGATE using UltraCamXp images. The height variation in UAV point clouds were similar on field, grass and gravel, while on the very homogeneous asphalt road the height variation was slightly larger due to matching failures. The point clouds provided by Photosynth were clearly noisier: the height variations were 0.3–0.6 m on planar objects. The higher noise level could be caused, on one hand, by the less accurate matching method (feature based matching) and, on the other hand, by the less accurate exterior orientations.

**Figure 9.**
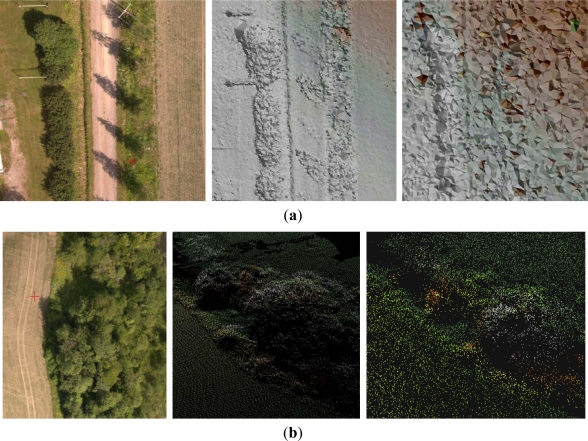
Examples of point clouds: (**a**) Gravel road (shaded representation) and (**b**) low vegetation. **Left**: GF 1 image; **center**: NGATE; **right**: Photosynth.

Some details of the point clouds are shown in Figure 9. As could be expected based on the previous results, the NGATE point cloud had a low internal deviation in flat areas while the Photosynth point cloud was clearly noisier (Figure 9(a)). Detailed evaluation of the NGATE point cloud showed that there were height artifacts in flat areas that were shadowed by trees. The single trees appeared in the Photosynth point cloud but not in the NGATE point cloud. The NGATE managed to measure the clothes rack that appears in the upper left corner in the image. The examples of point clouds in areas with low vegetation in Figure 9(b) show very good quality with both methods.

**Figure 10.**
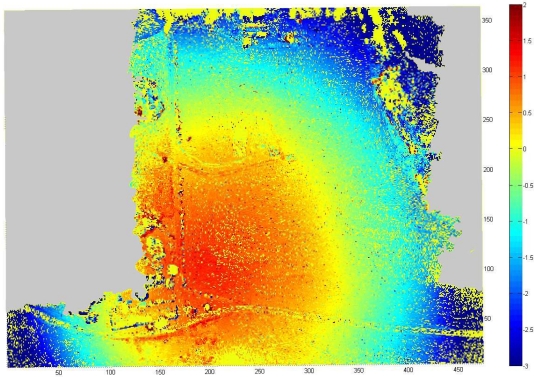
Differences between Photosynth and reference DSMs with 1 m × 1 m point spacing.

**Figure 11.**
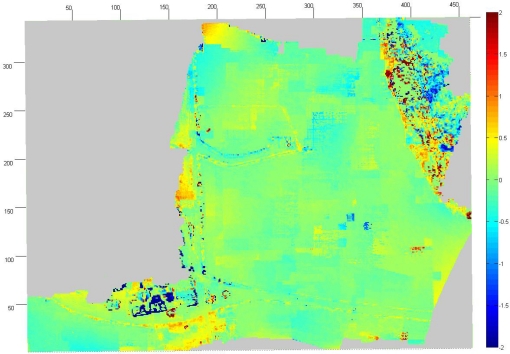
Differences between NGATE and reference DSMs with 1 m × 1 m point spacing.

Absolute accuracy of the UAV point clouds was studied using the UltraCamXp point cloud as a reference (estimate of σ_obj(Z)_). We sampled the point clouds to a 1 m × 1 m grid and calculated the differences to the reference point cloud. The point cloud by Photosynth was heavily distorted with a ball shaped distortion (Figure 10). In the center area, the Photosynth model was approximately 1.5 m above the reference, and, at the edge, it was approximately 3 m under the reference; RMSE was 1.5 m over the whole area. The accuracy of the point cloud provided by NGATE was clearly better with an RMSE of 0.15 m (Figure 11). The result is consistent with the value based on error propagation (combination of σ_isec(Z)_ and σ_def(Z)_). Also some small systematic deformations appeared in the NGATE point cloud, which appeared as edges following flight lines and stereo pairs throughout the block area.

**Figure 12.**
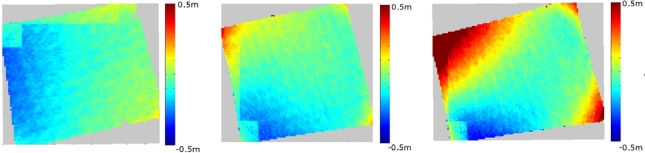
Differences of DSMs calculated from stereo models with 60% (**left**), 80% (**center**) and 90% (**right**) overlaps to the reference model.

We tested also the effect of stereo overlap percentage on the accuracy of point cloud by NGATE on single stereo models. The theoretical estimate of the height error of the stereo model measurement with a parallax measurement accuracy of GSD/2 is 4.9 cm, 9.8 cm and 19.6 cm for the stereo-overlaps of 60%, 80% and 90%, respectively (Table 5) [25]. Point clouds were calculated from the stereo models over a flat field area with 60%, 80%, and 90% overlap percentages using NGATE. All point clouds were sampled to a 1 m × 1 m grid, and the differences to a reference point cloud were calculated (Table 5, Figure 12). The point cloud derived from the stereo model with a 60% overlap provided the smallest random height difference of 8.5 cm to the reference. The point cloud derived with an 80% overlap provided almost as high accuracy as the 60% overlap. As expected, the 90% overlap had the highest random height error of 20.8 cm and showed large deformations at the edge of the stereo model. It is likely that the best possible accuracy with a field object is about 10 cm, which would explain the poorer than expected accuracy of the 60% data.

### 4.4. Masala Results

**Figure 13.**
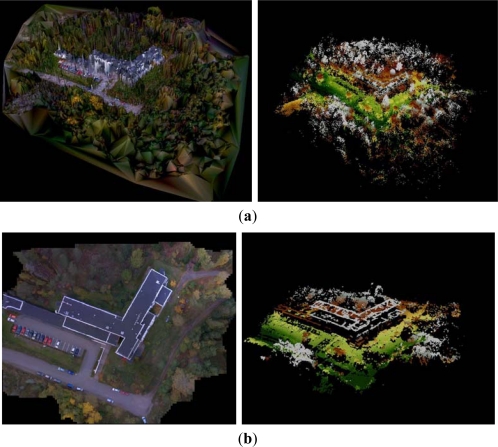
Point clouds from the FGI main building provided by (**a**) Photosynth (**left**: RGB, **right**: earth tones) and (**b**) NGATE (**left**: image mosaic, **right**: point cloud in earth tones). In point clouds the black areas are without points.

Photosynth managed to orient all the images (oblique and nadir) in a local coordinate system. The point cloud is shown in Figure 13(a). Eight corners of the building were identified from the Photosynth point cloud and the parameters of the 7-parameter similarity transform were calculated. After the least squares adjustment, the RMSE was 0.599 m and the standard deviation of unit weight was 0.712 m. Parameters were used to transform the point cloud to global coordinate system.

Only the nadir images were processed in SOCET SET. We used 81 uniformly distributed tie points per image and determined the initial approximations of exterior orientations by estimating the positions of image centers in an orthophoto. Statistics of self-calibrating bundle block adjustment indicated good orientation accuracy with standard deviations (RMS value) of 1.9, 2.4 and 1.4 cm for the perspective center positions in X, Y and Z directions, respectively, and 0.020°, 0.025° and 0.006° for rotations ω, φ and κ, respectively. The standard deviations (RMS value) of point unknowns were 2.2, 1.5 and 5.7 cm in X, Y and Z coordinate, respectively. The control points were used as checkpoints iteratively (one at a time) and the RMSE was 8.0, 8.0 and 6.7 cm in X, Y and Z coordinate, respectively. Difference between theoretical and empirical values is partially caused by the uncertainty in GCPs; systematic errors are likely to be low in this case. A point cloud was created by NGATE with a 10 cm point interval using the default strategy ngate_urban_strategy (Figure 13(b)).

Visual evaluation indicated that the NGATE and Photosynth point clouds differed in many areas. NGATE produced a point cloud with less noise than the one produced by Photosynth. On the other hand, NGATE clearly failed in the matching of tree surfaces in forest areas where Photosynth was more successful. Both point clouds failed with monotonic surfaces, *i.e.*, roof tops and the asphalt road.

We carried out detailed evaluations of point clouds in different objects in windows of the size of 3 m × 3 m (Table 6). A total of 24 sample squares were selected: 9 in forest, 9 in grass and 6 in asphalt. The Masala area was significantly smaller than the Sjökulla area, which lead to a smaller amount of sample windows. The success rate in Masala was clearly smaller than in Sjökulla. The only exception was Photosynth’s success with the ‘grass’ class with a point density of 14.9 points per m^2^, which clearly differs from all other classes having point densities of 0.6–2.1 points per m^2^. With NGATE, in the building, the point cloud generation was successful only on the edges of the roof, where edge based matching were used. The same problem occurred with road surfaces. The Photosynth point cloud had less than one point per square meter and the NGATE point cloud had less than three points per square meter on road surfaces. The performance in the homogenous surfaces in the Masala data being worse than in the Sjökulla data is most likely due to worse image quality; Ricoh images appear to be more noisy than Lumix images. Other reasons for the worse performance could be that the surfaces in Masala are even more homogenous than the surfaces in Sjökulla and Masala’s poorer weather and illumination conditions.

We could not evaluate the interior accuracy in these windows. The only flat surfaces in the area were in both point clouds mainly without points.

## 5. Summary and Recommendations

### 5.1. General Results

This article investigated point cloud generation from airborne image sequences collected with light UAV quadrocopter systems. The entire measurement system consisted of an UAV imaging system, data collection process and image data post-processing. The approach is suitable for applications which do not require real-time response. Two different hardware setups were tested: Panasonic Lumix GF1 mounted on Microdrone md4-1000 and the Ricoh GR3 compact camera mounted on Microdrone md4-200. The advantage of this UAV type is that it allows flexible data collection scenarios with adjustable flying speed and various data collection modes. This is well-suited for accurate measurement of objects and for evaluation and development of different experimental sensors. The performance of two different post-processing approaches was investigated: a traditional photogrammetric processing based on Bae Systems’ SOCET SET and an Internet freeware processing built around Microsoft’s Photosynth service. The systems were tested in two test areas.

In both missions, Photosynth was able to orient all gathered images, both vertical and oblique, in a local coordinate system without a priori orientation information. This means that it is possible to carry out aerial photogrammetric processing using a light UAV and a standard camera without GNSS and IMU support and only relying on image matching; good results of a processing line for UAV images based on invariant features was also previously reported in [43]. Photosynth determined sensor calibration separately for each photo, which is a feasible approach for image sets gathered with multiple cameras and zoom lenses. In our application, the approach was not optimal, because the camera system had a fixed focal length and focus was set to infinity. The solution was non-stable with a correlated principal distance and the exterior orientation height coordinate; this could be seen especially in the Sjökulla mission where the object was flat and where only vertical images were used. The point cloud calculated by Photosynth showed promising results. Even though its point densities were not as high as with traditional photogrammetric software and the final DEM was distorted with a ball shape distortion and burdened with noise, it managed to produce a point cloud with an accuracy of 1–3 m when transformed to the desired object coordinate system using control points, and thus the point cloud represented the object of interest mostly in right shape. It is important to bear in mind that Photosynth is intended for photocollection applications and most likely not optimized for point cloud generation; the Microsoft’s Geosynth could be more appropriate for point cloud generation [39]. A further complication was that with the Photosynth there were many unknown components in the process, and it was not possible to rigorously check the consistency with respect to theoretical expectations.

Processing by SOCET SET provided dense and accurate point clouds. With the Lumix GF 1 images from the Sjökulla mission NGATE managed to produce a point cloud that was almost as good in point density and height accuracy as the reference point cloud provided by the large-format photogrammetric camera. On the other hand, low altitude and large three-dimensionality caused problems that showed as failed matching, especially in forested areas. This may have something to do with the fact that SOCET SET was designed for traditional aerial photogrammetry and not for photogrammetry with a low flying UAV and drastic perspective differences between images. Further improvement in its performance could be obtained by optimizing the parameters or by using seed height models. The results of the photogrammetric processing by the SOCET SET followed very well the theoretical expectations based on error propagation, which can be considered as a validation of the entire processing line for metric purposes. The basic constraints related to atmospheric conditions, illumination, image quality and geometric relationships caused predictable variation in point cloud quality.

Our post-processing requires some further development in order to be fully autonomous. The most critical issue is the determination of approximate orientations. The direct exterior orientations provided by Microdrones were not accurate enough in the preliminary tests, so we determined approximate orientations interactively. We expect that novel ordering methods (such as Photosynth) would be operational in providing approximate orientations, but we have not yet integrated Photosynth and SOCET SET. Also other alternatives, such as the method for automatic production of orthophoto mosaics from video flow developed by Zhou [9,10], would most likely be appropriate for providing orientations.

The completeness and accuracy of image matching is highly dependent on image quality, object and matching method as discussed in Section 2.4. In the empirical evaluations, these issues appeared as follows:

1. **Image quality**. In this study, image quality varied from poor (Ricoh images collected under clouds in Masala) to good (Lumix images collected in sunny weather in Sjökulla). The greatest difficulties related to image quality with both post-processing methods were experienced with the Masala data. With the Sjökulla data, we detected problems in areas shadowed by trees; this issue requires further considerations for many applications because shadows are likely to appear in UAV image data. Also, images were not collected in windy conditions, which might be a further issue decreasing quality.
2. **Object**. In this study, problems were detected in matching of thin objects (electric poles), vague objects (trees) and homogeneous surfaces (FGI building roof, asphalt). In textured field surfaces the matching could be carried out with very good success. We expect that many of the problems experienced could be solved by using optimal data collection (especially the use large forward overlaps) and by improving matching strategies.
3. **Matching method**. Matching method related factors, together with the orientation accuracy, most likely caused the differences in the results of Photosynth and SOCET SET observed in this study. We expect that further tuning of the processes will improve performance of both approaches.

It is important to be aware of these factors and to understand their interactions and their influences in final applications. This is a relevant issue with UAV systems, because the images are likely to be collected in deviating conditions using a wide variety of sensors.

Different applications will have different requirements. This investigation considered point cloud generation in a relatively general level. We were able to demonstrate that many issues influence performance, and different objects have different performance. In specific applications, it will be necessary to go into more details of objects of interest, and find appropriate GSDs, flying speed, wind limits and other parameters, and quality indicators for UAV missions.

### 5.2. Recommendations

Based on the investigation, we give the following recommendations for data collection and orientation processing from the point of view of accurate point cloud generation by quadrocopter type micro UAVs equipped with a single camera.

The images should be collected with high forward overlaps, at least 80–90%. In our case, the ideal approach is in most cases a preprogrammed flight with continuous shoot mode, whereas for detailed measurements, the manual mode with a live video feed with virtual glasses has proven to be functional.

Careful orientation processing is necessary to obtain reliable results. High stereo overlaps should be used; increasing the overlap from 80% to 90% clearly improved the accuracy. The self-calibrating bundle block adjustment approach is recommended because the system calibration can be unstable.

When a camera is used as a measurement tool, it is important to keep in mind that quadrocopter type UAVs are highly unstable as camera platforms due to vibration caused by motors and propellers and drastic changes in movement speed. A stable and firm lens should be used and it should keep its calibration well. Therefore, pocket size cameras that have lenses that retract in a moment of shutdown should be avoided in campaigns that require high accuracy measurements. Moreover, we have found out that exposure times as short as 1/2,000 s are required for constantly obtaining pictures without motion blur. High frame rates are required to provide high forward overlaps. High dynamic range is a preferred quality in a camera, because photos should have well identifiable information in shadowed areas without causing saturation in the bright areas. Therefore, large physical pixel size is a desired feature for a mapping camera. Ricoh GR3 was not ideal with respect to these recommendations. The images obtained by it appeared to be of poor quality, which can be one reason for the poor quality of point clouds in Masala. Lumix GF1 provided higher quality images and also the quality of point clouds was better. To improve the quality of matching with lowest-weight systems, higher quality low-weight imaging sensors need to be developed.

The importance of ground control data in local area remote sensing applications should be emphasized here. GNSS/IMU observations are helpful in providing approximate orientations, but typically integrated sensor orientation with self-calibration is recommended, due to low quality of available low-weight GNSS/IMU systems and due to instability of system calibration [9,10]. However, as the simulation study showed, even the relatively low accuracy direct orientation observations can improve the absolute georeferencing accuracy; with high quality GNSS or GNSS/IMU data, in theory, there is no need for GCPs at all. The empirical study demonstrated the use of various reference materials, including targeted GCPs, existing high-resolution photogrammetric data and details measured in an object by VRS GNSS. Especially in the case of VRS GNSS measurement, we experienced difficulties in measuring corners of roofs in field and in identifying the corners in images, because the corners were ambiguous and exact measured positions were not documented. In any case, the number, distribution and accuracy of GCPs do influence the accuracy of georeferencing.

We recommend the development of orientation methods for unordered image sequences. This allows data processing that is the most reliable, also in cases of failures with the GNSS/IMU systems. In our study, the results with the Microsoft’s Photosynth service proved that these systems can order image sequences and produce relatively good-quality point clouds; the highest accuracy can then be obtained by rigorous photogrammetric processing. The future environmental measurement systems will combine different instruments [17,18], but also with these systems rigorous processing of data sets collected by individual sensors will be relevant. An operational method for accurate image based point cloud generation would combine an approximate method (such as Photosynth) with the highest accuracy methods (such as NGATE); in this study we did not integrate these methods. We also recommend continuation of development of accurate, low-weight GNSS/IMU systems, as accurate orientation information will simplify the processes and improve the accuracies greatly. Most likely the optimal method for many applications in the future will be a tightly integrated multisensorial system similar to the one developed by Nagai *et al*. [17].

All imaging systems and block set-ups will have their limitations, and simulation is a powerful tool for examining these limitations. We suggest that in the UAV applications with high point determination accuracy requirements it is important to ensure that the data theoretically fulfils the requirements, because data collection scenarios can vary a lot, and also failures can appear in the data set. Necessary steps are a design of the image block, careful collection of data and quality control after the image collection. Comparisons of theoretical and empirical results will give further insight to the quality of the imaging system and processing. In our case, the empirical results were at best only slightly poorer than our theoretical expectations; this indicated that there were only small, remaining image distortions.

Overall, this investigation provided very promising results with the UAV based aerial photogrammetry. This is a fascinating field of remote sensing, offering large variety of options and many new applications but also requiring very rigorous processing in order to provide controlled results.

## Figures and Tables

**Table 1.** Interior orientation and radial distortion parameters by iWitness, SOCET SET and Photosynth. Principal point is given from the image center. For the SOCET SET processing also the standard deviations are given.

**Camera**	**Software**	**c (mm)**	**x0; y0 (mm)**	**k1**	**k2**	**k3**
Lumix GF1	iWitness	20.099	−0.119; 0.193	5.02E−04	1.06E−06	−8.01E−09
	SOCET SET	-	−0.002; −0.042	5.54E–04	−1.80E–07	3.47E–10
	stdev	-	0.003;0.003	2.78E–06	6.52E–08	4.70E–10
	Photosynth	20.20	-	-	-	-
Ricoh GR3	iWitness	5.953	0.024; 0.034	1.81E–03	−5.94E–05	5.27E–07
	SOCET SET	5.945	−0.015; 0.067	1.71E–03	−6.52E–05	1.05E–06
	stdev	0.009	0.004; 0.007	4.83E–05	4.35E–06	1.84E–07
	Photosynth	6.13	-	-	-	-

**Table 2.** Points per square meter and relative point densities evaluated in different objects in 3 m × 3 m areas. The point clouds were from UltraCamXp by NGATE (10 cm point interval), GF1 by NGATE (5 cm point interval) and GF1 by Photosynth.

	**Points per square meter**					**Relative point density**				

	**Field**	**Grass**	**Forest**	**Asphalt**	**Gravel**	**Field**	**Grass**	**Forest**	**Asphalt**	**Gravel**
UC NGATE	82.1	86.9	47.6	89.6	91.9	0.82	0.87	0.48	0.90	0.92
GF1 NGATE	278.2	361.8	136.0	233.0	304.4	0.70	0.90	0.34	0.58	0.76
Photosynth	2.9	2.4	2.1	2.4	3.5	-	-	-	-	-

**Table 3.** Point determination accuracy estimates. The adjustment cases are the simulated case and the empirical evaluations with self-calibration (Self-calib) and without self-calibration (No self-calib).

**Case**	**σ_0_ (pixel)**	**Theoretical (cm) Standard deviation**			**Empirical (cm) RMSE**			**Standard deviation**		

		**X**	**Y**	**Z**	**X**	**Y**	**Z**	**X**	**Y**	**Z**
Simulated	1.00	2.9	2.7	6.2	-	-	-	-	-	-
Self-calib	0.87	0.7	0.8	3.1	2.9	4.3	10.6	2.9	2.5	8.2
No self-calib	5	0.6	0.7	2.7	23.7	16.9	518.0	17.0	15.0	170

**Table 4.** Random height variations evaluated in different objects in 3 m × 3 m areas. UltraCamXp processing was carried out with NGATE with a 10 cm point interval and the GF1 point cloud was created using NGATE with a 5 cm point interval and using Photosynth.

**Material**	**Random height variation (m)**				
	**Field**	**Grass**	**Forest**	**Asphalt**	**Gravel**
UC NGATE	0.08	0.11	0.47	0.10	0.10
GF1 NGATE	0.10	0.09	0.87	0.16	0.12
GF1 Photosynth	0.29	0.31	0.67	0.57	0.33

**Table 5.** Effect of the overlapping percentage in height accuracy of DSMs provided from stereo models with 60%, 80% and 90% stereo overlaps by NGATE.

**Overlap (%)**	**Theoretical (m)**	**Random (m)**	**Systematic (m)**
60	0.049	0.085	−0.096
80	0.098	0.097	−0.060
90	0.196	0.208	0.032

**Table 6.** Points per square meter and relative point densities (Equation (2)) in 3 m × 3 m areas in the point clouds created from Ricoh GR3 images using NGATE (a 10 cm point interval) and Photosynth.

	**Points per square meter**			**Relative point density**		

	**Grass**	**Forest**	**Asphalt**	**Grass**	**Forest**	**Asphalt**
GR3 NGATE	45.4	21.3	2.8	0.45	0.21	0.03
GR3 Photosynth	14.9	2.1	0.6	-	-	-
